# The Association of Post-Separation Abuse and Coercion With Children’s Health and Flourishing

**DOI:** 10.1111/jan.70681

**Published:** 2026-07-17

**Authors:** Kathryn J. Spearman, Nancy Perrin, Christina Bethell, Kamila A. Alexander, Jennifer Hardesty, Jacquelyn Campbell

**Affiliations:** 1 Ross and Carol Nese College of Nursing and Child Maltreatment Solutions Network, The Pennsylvania State University, University Park, Pennsylvania, USA; 2 Johns Hopkins University School of Nursing, Baltimore, Maryland, USA; 3 Johns Hopkins University Bloomberg School of Public Health, Baltimore, Maryland, USA; 4 University of Illinois Urbana-Champaign, Urbana, Illinois, USA

**Keywords:** children’s health, coercive control, intimate partner violence, paediatric access to health care, post-separation abuse, unmet health needs

## Abstract

**Aim:**

The purpose of this study is to compare health outcomes and flourishing between children (age 6–17) whose mothers experience high versus low exposure to post-separation abuse and coercive control (PSA).

**Design:**

Exploratory sequential mixed-methods study.

**Methods:**

Cross-sectional survey with community-based, convenience sample of family court-involved mothers in the United States (*N* = 497). Independent variable measured by subscales of the Healthcare, Economic, and Legal PSA and Coercive Tactics Scale (HELP-T). Health outcomes measured by items from National Survey of Children’s Health.

**Results:**

Children whose mothers experience more frequent PSA face a dual burden: increased odds of unmet mental health needs coupled with increased odds of mental health diagnoses, somatic symptoms and special health care needs. Children in the high-exposure Healthcare PSA group were twice as likely (aOR: 2.01, *p*-value < 0.001, 95% CI: 1.4–2.9) to report stomach/intestinal problems, 2.3 times more likely (*p*-value < 0.001, 95% CI: 1.5–3.4) to report chronic pain including headaches in the last 12 months, 2.13 times more likely (*p*-value < 0.001, 95% CI: 1.4–3.8) to have ever received a diagnosis of anxiety and 2.72 times (*p*-value < 0.001, 95% CI: 1.60–4.63) more likely to have unmet mental health needs as compared to children in the low-exposure group.

**Conclusion:**

The findings from this study advance the growing body of evidence on how co-parent perpetrated PSA and coercive control harm children’s health and access to healthcare.

**Impact:**

This study is the first to our knowledge to demonstrate the association between maternal exposure to co-parent perpetrated PSA and children’s health outcomes, establishing a foundation for future intervention work to improve children’s health and access to health care.

**Public Contribution:**

Members of the public were involved in recruitment and cognitive interviewing.

## 1 | Introduction

Child health is profoundly impacted by exposure to intimate partner violence (IPV). Approximately 15 million children in the United States are exposed to IPV each year, with up to one in four children exposed to IPV prior to age 18 (Finkelhor et al. 2015; McDonald et al. 2006). Children exposed to IPV also tend to experience more severe maltreatment and injury than children not exposed, as perpetrators often coerce and assault multiple family members (Katz et al. 2020; MacMillan and Wathen 2014; Stark 2023). Childhood exposure to IPV frequently intersects with other adverse childhood experiences (ACEs), such as parental separation and divorce. About one in three American children will experience parental separation, and about one in four divorces are attributed to IPV (Beck et al. 2013). Social and environmental factors such as exposure to IPV and other relational health risks are important social determinants of health across the lifespan.

Childhood IPV exposure is an environmental threat associated with disruptions in neurobiological, cognitive and emotional developmental processes (McLaughlin et al. 2016). Throughout this manuscript we use the term ‘exposure’ in the epidemiological sense; however, this should not be interpreted as children being passive witnesses to IPV. Children experience and resist IPV and regimes of coercive control with all their senses, and these emotional and physical experiences become embodied (Callaghan et al. 2016). Threat, or fear, is caustic to the child’s developing brain and body (Fox and Shonkoff 2012). Children exposed to IPV have higher rates of depression, anxiety, use of psychotropic medications, internalizing and externalizing behaviours, obesity and asthma compared to children not exposed (Bair-Merritt et al. 2015; Berg et al. 2022; Holmes et al. 2022a, 2022b). This human suffering also exacts an economic toll. Childhood exposure to IPV is a significant economic burden to society, estimated to be $55 billion for an annual birth cohort in the United States (Holmes et al. 2018). This increased economic burden is largely attributed to increased healthcare costs, lost productivity and increased crime costs over the lifespan (Holmes et al. 2018). Holmes et al. (2018) estimated that the average lifetime cost per child exposed to IPV is over $50,000 in 2016 dollars, with $11,000 of this total per child survivor due to increased healthcare costs. Fang et al. (2012) estimate that per nonfatal child maltreatment case is $210,012 in 2010 dollars, including $32,648 in childhood health care costs; $10,530 in adult medical costs. Estimates of total lifetime costs of new childhood abuse and neglect cases is estimated to be between $124 and $500 billion (Fang et al. 2012). These are likely substantial underestimates.

A growing body of evidence has highlighted the strong association between childhood exposure to IPV and adverse physical health (Bair-Merritt et al. 2006, 2015; Bender et al. 2022; Gartland et al. 2021; Holmes et al. 2022a, 2022b), mental health (Campbell et al. 2021; Greene et al. 2018; Miller-Graff and Howell 2015), paediatric health care needs and access (Bair-Merritt et al. 2008; Blake et al. 2026; Holmes et al. 2022a, 2022b; King and Khanijahani 2020), social and behavioural outcomes (Galano et al. 2022) and academic outcomes (Cage et al. 2022). However, substantial gaps in knowledge remain.

Most of the existing studies that have examined the association of children’s exposure to IPV have measured IPV with a single item, usually assessing only witnessing physical IPV (e.g., how IPV is measured in the National Survey of Children’s Health [NSCH]) (Child and Adolescent Health Measurement Initiative [CAHMI] 2025), or use measures of IPV such as the Conflict Tactics Scale (Straus et al. 1996), which many scholars have argued does not capture coercive and controlling behaviours that are central to the most persistent and severe forms of domestic violence (Johnson 2006). Additionally, few measures of IPV differentiate between abuse that was perpetrated by a current intimate partner versus a former intimate partner (e.g., capturing abuse that occurs following separation). Measures of IPV that incorporate more nuanced coercive or abusive behaviours across domains and include ways in which children are affected by coercive, controlling or abusive tactics by perpetrators are needed to extend knowledge about the multifaceted ways in which IPV is detrimental to children’s health.

Studies on children’s health in the context of IPV have recruited from different populations: shelter-based, community settings and clinical settings. Across these samples, scholars have found similarly strong associations between IPV exposure and adverse child health. Most of these studies have been cross-sectional; however, a few longitudinal studies that identify the timing of exposure and later developmental outcomes (Gartland et al. 2021) strengthen our understanding of the association between IPV and adverse child health outcomes. Yet none of these studies, to our knowledge, have examined differences in IPV exposure for family court-involved children whose parents have separated/divorced in the context of IPV. This distinction is important because of legal contexts that regulate shared parenting. Differential interventions and thoughtful adjudication are needed to promote child health in the context of ongoing IPV and coercive control following parental separation/divorce (‘post-separation abuse’) for family court and child welfare involved children (Spearman, Marineau, et al. 2025).

Separation is commonly assumed to be the solution for adult survivors to obtain safety, but this does not guarantee child safety. Nor does separation guarantee safety for the adult survivor: substantial evidence supports the increased risk of lethality in the context of separation (Campbell et al. 2003), as well as increased physical violence (Rezey 2020) and the emergence of other forms of abuse such as legal abuse (Gutowski and Goodman 2023). And even if physical proximity has been limited when co-parents are no longer cohabitating, thus reducing potential exposure to physical or sexual IPV, little is known about how ongoing emotionally abusive or coercive IPV behaviours impact child health outcomes or access to healthcare.

Despite the millions of American children who are both exposed to IPV and experience parental separation/divorce during their childhood, this group of children has been relatively neglected in the scientific literature on children’s health. Research on the impact of post-separation abuse on children’s health is needed to address three critical gaps. First, existing research has relied predominantly on measures of physical IPV that fail to capture the coercive and controlling behaviours that are central to the most persistent abuse. Second, virtually no studies have examined children’s health in the post-separation period specifically, despite evidence that separation does not end, and may even exacerbate, abuse. Third, family court-involved children, who face legally mandated ongoing contact with an abusive parent, represent a distinct and understudied population whose health needs are largely invisible to both medical and legal systems. This study addresses all three gaps simultaneously by examining how specific co-parent perpetrated PSA tactics, as measured by the novel HELP-T (Healthcare, Economic, and Legal Post-separation abuse and coercive Tactics) subscales, are associated with children’s health outcomes, access to care and flourishing in a family court-involved sample.

Informed by the IPV and Co-parenting After Separation (ICAS) Model, a conceptual framework developed by Hardesty et al. (2012), the aim of this study is to compare children’s health and flourishing outcomes between children whose family court-involved mothers who experienced high versus low exposure to post-separation abuse and coercive control (PSA) in the past 12 months. The ICAS Model (Hardesty et al. 2012) identifies risk and protective factors for co-parenting violence, coercion and conflict after separation and the association with maternal and child physical and mental health outcomes. For this study, we operationalized the co-parenting/post-separation relationship outcome domain of the ICAS model as PSA, measured by the Healthcare, Economic and Legal PSA subscales of the HELP-T (Spearman, Perrin, et al. 2025). For children’s health outcomes, we measured children’s special health care needs (CSHCN), unmet mental health needs, flourishing, two functional difficulties in the past 12 months (stomach problems, chronic pain including headaches) and ever receiving a diagnosis of depression or anxiety.

## 2 | Study Design

The present study used a cross-sectional survey design with a community-based convenience sample and presents integrated mixed-methods data from an exploratory sequential mixed-methods research study, a study design that is best suited for exploring a phenomenon when existing literature is inconclusive or lacking (Creswell and Plano-Clark 2018). Consistent with the exploratory sequential mixed-methods design, qualitative interviews were conducted first to inform the development of the cross-sectional survey for the quantitative phase.

## 3 | Methods

### 3.1 | Qualitative Sample, Data Collection and Analysis

Thirty-three community-based maternal survivors of post-separation abuse and coercive control from 18 states in the contiguous United States participated in semi-structured interviews from January through June 2023 on their experiences of post-separation abuse and coercive control, family court and their children’s health. Most (*n* = 30) qualitative participants were within 2 years of separation. Interviews ranged from 47 to 246 min, with some interviews conducted over two sessions. All transcripts were double coded by three members of the study team using codes from a codebook developed a priori, and iteratively refined as new codes emerged. This paper integrates quotes from the code group for child health outcomes, including codes for several subdomains: behaviour, dental, health insurance, mental and emotional, nutrition, physical and somatic, special health care needs, sleep, schooling, unmet health needs and flourishing. In this paper, quotes using participant pseudonyms from the qualitative arm of the study were selected by code for each subdomain. Quotes are used illustratively alongside quantitative findings to centre the lived experiences of survivors and contextualize the statistical associations for each child health outcome examined by the study team. Further details on interview guide and codebook development, recruitment and sampling strategies, cognitive testing and qualitative data analysis are reported elsewhere (Spearman et al. 2024, 2026; Spearman, Marineau, et al. 2025).

### 3.2 | Quantitative Sample and Data Collection

Mothers of children ages 6–17 in the United States were recruited from May 2024 through August 2024 through social media and snowball sampling (word of mouth from other participants) to respond to a survey about demographic items, court involvement, measures on their children’s health and well-being, experiences of IPV, housing instability and items on the HELP-T (author citation). Participants received a link to an anonymous survey through a QR code on flyers to complete the online survey through REDcap (Research Electronic Data Capture). Flyers advertised the opportunity to participate in research about experiences of seeking child custody following separation from an ex-partner, and how children are doing. Flyers were distributed primarily through social media by domestic violence advocates, legal aid organizations and other survivors through social media private support groups (e.g., support groups for single mothers). Participants could directly access the anonymous survey and complete it through a public link. Some participants contacted the first author seeking more information and received an individualized link. Survey responses were verified through a protocol developed to eliminate bot and fraudulent responses. Participants were contacted by phone or Zoom following the survey to verify authenticity of responses. Verified participants received a $10 gift card for their time. In total, the final quantitative sample included *N* = 497 maternal participants from 47 US states and Washington, DC. The three states with the most participants were California (*n* = 53, 10.7%), Pennsylvania (*n* = 29, 5.8%) and Colorado (*n* = 26, 5.25%).

Inclusion criteria included mothers who (1) were over the age of 18, (2) had a child between the ages of 6 and 17 that they share with a co-parent they have separated from, (3) identified as feeling afraid for themselves or their children since separation, and (4) have experienced post-separation abuse from their co-parent and/or have an active family court case in the last 12 months. Exclusion criteria include mothers who (1) do not have access to their children’s health information, (2) do not live in the United States, or (3) do not co-parent with the child’s other parent (e.g., child’s other parent is no longer living, or they co-parent with another relative who was not an intimate partner like a grandparent).

### 3.3 | Ethical Considerations

Ethical approval was obtained from the Johns Hopkins Institutional Review Board (IRB00336841) on December 22, 2022. Prior to completing an online survey, participants reviewed key information about the study and indicated that they were willing to participate.

### 3.4 | Measures

#### 3.4.1 | Post-Separation Abuse and Coercive Control

The independent variable of interest was maternal experiences of post-separation abuse and coercive control perpetrated by co-parents, as measured by the HELP-T (author citation). The HELP-T consists of three subscales: Healthcare, Economic and Legal PSA. Subscale scores are calculated as the mean of item responses (row means), with higher scores indicating greater frequency of exposure. Each subscale of the HELP-T assesses the frequency of co-parent behaviours using a Likert scale, ranging from 1 (not in the last 12 months), 2 (once), 3 (a few times), to 4 (many times) over the past 12 months. To operationalize PSA, we split each of the subscales of the HELP-T (Healthcare, Economic, Legal PSA) into high and low groupings based on the median value for each subscale (Table 1). Participants who scored above the median for each subscale were placed into the ‘high-exposure group’ and participants who scored below the median were placed in the ‘low-exposure group’. Given that the HELP-T is a new measure and has not yet been validated in diverse clinical or community samples, the median score was chosen as the threshold for groupings. Importantly, ‘low exposure’ in the past 12 months does not mean no exposure: all participants were family court involved, endorsed ever experiencing PSA since separation from their co-parent, and all but two participants (who had non-zero, subthreshold IPV scores) were IPV-positive based on the Composite Abuse Scale—Short Form developed by Ford-Gilboe et al. (2016).

#### 3.4.2 | Health Outcomes

The following health variables of interest, which are items in the National Survey of Children’s Health (NSCH) (CAHMI 2025), were examined as outcome variables to assess the relationship between PSA and children’s health: (1) currently meets children with special health care needs (CSHCN) status, (2) functional difficulties during the last 12 months including stomach or intestinal problems and chronic pain; (3) ever receiving a diagnosis of depression or anxiety; (4) inability to obtain mental healthcare during prior 12 months; and (5) currently meets criteria for flourishing. Maternal participants with more than one child were asked to respond to the items selecting the child they thought was most affected.

The CSHCN measure is based on the five question NSCH screener to operationalise the children with special health care needs definition from the US Census Maternal and Child Health Bureau. The CSHCN focuses on health consequences rather than a specific diagnosis or disability (Bethell et al. 2015), and asks about children’s current (1) use or need of prescription medication; (2) above average use or need of medical, mental health or educational services; (3) functional limitations compared with others of the same age; (4) use or need of specialised therapies (e.g., occupational therapy [OT], physical therapy [PT], speech); (5) treatment or counselling for emotional, behavioural or developmental problems. For each of these five questions, the screener asks those who respond yes whether the consequence is due to any kind of health condition and, if so, whether that condition is expected to last for at least 12 months. All three parts of at least one screener question must be answered ‘yes’ in order for a child to meet the criteria for CSHCN.

To operationalize functional difficulties experienced by children in the last 12 months, we administered two items from the NSCH: (i) difficulty with digesting food, including stomach/intestinal problems, constipation or diarrhoea; (ii) difficulty with repeated or chronic physical pain, including headaches or other back or body pain.

We assessed whether children had ever been diagnosed with depression and anxiety using items on the NSCH (CAHMI 2025): ‘Has a doctor or other health care provider EVER told you that this child has depression?’ and ‘Has a doctor or other health care provider EVER told you that this child has anxiety problems?’ If yes, participants were asked if the child currently had the condition and if it was mild, moderate or severe.

To operationalize unmet mental health needs, we administered two items from the NSCH (CAHMI 2025) asking first ‘During the past 12 months, has this child received any treatment or counseling from a mental health professional?’ with responses (1) Yes, (2) No but this child needed to see a mental health professional, and (3) No this child did not need to see a mental health professional, and secondly, ‘How difficult was it to get the mental health treatment or counseling that this child needed?’ with responses on a Likert scale from (1) Not difficult to (4) It was not possible to obtain care. This variable was dichotomized, with responses of ‘It was not possible to obtain care’ coded as unmet mental health need. Children whose mothers responded ‘no, this child did not need to see a mental health professional’ to the first item were not included in the analysis of unmet mental health needs.

Flourishing is a composite measure based on three items from the NSCH (CAHMI 2025): ‘How often does this child: (i) *show interest and curiosity in learning new things*, (ii) *work to finish tasks they start*, and (iii) *stay calm and in control when faced with a challenge*?’ Children with a response of ‘Usually’ or ‘Always’ to the questions indicate the child meets the flourishing item criteria. The variable is dichotomized to (1) met all flourishing items, or (2) did not meet all three flourishing items.

#### 3.4.3 | Covariates

We controlled for a common set of demographic and social health risk variables from NSCH items that were asked in our survey: child age; insurance type: Medicaid, private insurance; household ability to afford food; concerns for home eviction, receiving government assistance in last 12 months: cash assistance/welfare, SNAP benefits, receiving free or reduced cost breakfast or lunches at school, WIC or EBT cards; and family resilience. Family resilience is a composite NSCH measure based on four items: *(i) When you and your children face problems, how often are you likely to talk together about what to do?, (ii) When you and your children face problems, how often are you likely to work together to solve the problems?, (iii) When you and your children face problems, are you likely to know we have strengths to draw on?, (iv) When you and your children face problems, how often are you likely to stay hopeful even in difficult times?* Children were categorized as having family resilience based on maternal report if the responses were ‘All or most of the time’ to all four items. Family resilience has been shown to be protective for children exposed to adversity (Bethell et al. 2019).

### 3.5 | Statistical Analysis

All statistical analysis was run in Stata 17 (StataCorp 2021). Code for composite variables from the NSCH and for 2022–2023 NSCH (STATA dataset) analyses was used from the CAHMI Codebook (CAHMI 2025). For demographic, social health risk and health outcome variables, we conducted *t*-tests for continuous variables (e.g., child age) and chi-squared tests for binary and categorical variables between the low and high groups of each subscale. If these variables were significant (*p*-value < 0.05) in chi-squared tests between low-and high-exposure groups for any of the three subscales, they were chosen as potential covariates for the common model. Child sex, child Hispanic origin and maternal employment were not significant between the low and high groups of each of the three subscales, and were not included as covariates in the final adjusted models. We examined bivariate associations between demographic and social characteristics and health outcomes of interest. Family Resilience was significantly associated with flourishing (*β* = 1.22, *p*-value < 0.001), depression (*β* = −0.44, *p*-value < 0.017) and CSHCN (*β* = −0.44, *p*-value < 0.048), and was added as an as additional covariate in the adjusted models.

We then conducted bivariate logistic regressions to determine unadjusted odds ratios for each of the health outcome variables of interest with the low and high groupings of each of the three HELP-T subscales. Results of each of the three logistic regression models (Healthcare PSA, Economic PSA and Legal PSA) are reported as unadjusted and adjusted odds ratios with 95% confidence intervals with the low group for each subscale as the reference group. Verbatim quotes from participants in the qualitative arm of this study are integrated to contextualize quantitative findings for each of the health outcomes.

## 4 | Results

### 4.1 | Demographic and Social Characteristics of Sample

Selected child and household demographic and social characteristics as reported by maternal participants are reported in Table 2. Categories representing less than five participants have been collapsed to prevent possible identification and to protect participant confidentiality. The mean age of children was 11.2 (3.45) (Table 2). 11.9% of the sample identified the selected child as Hispanic, 10% of the sample identified the selected child as two or more races, 2.8% of the selected child sample identified as Black alone, and the majority of the sample identified the child as White alone (Table 2). 64% of the children were covered by private insurance, 29% were covered by Medicaid, and 4% were covered by other government insurance (Tricare or Indian Health Service). 53% (*n* = 265) of children are in a family (mother–child relationship) that demonstrates qualities of resilience. Mothers reported experiences of economic hardship: 21% of participant households could sometimes or often not afford enough to eat and 18.3% of participant households were always or usually concerned about being evicted. Nearly a third of participants (29%) received SNAP benefits, and 16.9% received cash assistance from the government (Table 2).

Participants experienced IPV from their co-parent as measured by items on the Composite Abuse Scale—Short Form (CAS-sf) (Ford-Gilboe et al. 2016) of having ever experienced that form of abuse from their co-parent and for their experiences in the last 12 months (Table 3). Nearly all participants (*n* = 495) were IPV positive as measured by the CAS-sf for the last 12 months, with only two participants scoring non-zero subthreshold IPV in the past 12 months. When asked about IPV exposure from their co-parent, most participants endorsed having ever experienced physical abuse from their co-parent, although rates were lower in the past 12 months. 27% of maternal participants (*n* = 134) endorsed that their co-parent had ever strangled them (Table 3). Over one-third (34%) of maternal participants reported that their co-parent had ever used or threatened to use a knife, gun or other weapon to harm them, and the rate was approximately 5% in the past 12 months (Table 3). Participants were also asked a number of items about their children’s unsafe exposures with their co-parent, and 33.2% of participants reported that their co-parent had put their child in situations that were unsafe or inappropriate with guns.

Participants reported a wide range of current legal and physical custody arrangements for their children, with the most common being joint legal (*n* = 130; 26.6%) and joint 50/50 shared physical custody (*n* = 238; 47.89%). 14.7% (*n* = 73) of participants reported that they had sole legal custody or decision-making; and 9% of participants (*n* = 44) reported that their co-parent had sole decision-making. 11.8% (*n* = 59) of participants reported that their co-parent had no contact or supervised contact; and 5.6% of participants (*n* = 28) reported that they had lost physical custody of their children after reporting abuse and had supervised or phone contact with their children only.

### 4.2 | Children With Special Health Care Needs

He seems very typical, but he really struggles in school, and so he has an IEP right now… In talking to my therapist, she’s like big on saying it’s because of the abuse, you know, the emotional and verbal abuse they experience at home could maybe contribute to some of their delays…He’s starting occupational therapy and speech therapy again through school.

—Edith, mom of two boys, ages 8 and 6

Three out of four children (78.9%; *n* = 392) within our overall sample have special health care needs (Table 4). For children who met CSHCN criteria, 36.2% qualified on prescription medicine, 54.9% met the qualifying criteria on elevated need of medical care, mental health or educational services, 26.2% met the qualifying criteria on need or use of special therapy such as occupational, speech or physical therapy, and 69.0% met the qualifying criteria on ongoing emotional, developmental or behavioural conditions.

Across all three subscales of the HELP-T, children whose mothers reported more frequent post-separation abuse were approximately two times more likely to meet CSHCN status as compared to children in the low group (Healthcare PSA: aOR: 2.27, *p*-value < 0.001, 95% CI: 1.41–3.65; Economic PSA: aOR: 1.94, *p*-value < 0.005, 95% CI: 1.22–3.07; Legal PSA: aOR: 2.05, *p*-value < 0.003, 95% CI: 1.30–3.30) (Table 5; Figure 1).

### 4.3 | Functional Difficulties

He was going to the school nurse quite often. He has a migraine. I’ve had to take them for migraines, and he says a lot of times that his stomach hurts. Which my understanding is stress. The exhaustion, the mental exhaustion.

—Joy, mom of 10-year-old boy

Functional difficulties are a broad measure within the NSCH of how a child’s health impacts their ability to participate in daily life. In this study, we focused on measuring two functional difficulties that were frequently mentioned by qualitative participants in the first phase of the study, including experiences of stomach aches or chronic pain including headaches in the prior 12 months.

#### 4.3.1 | Frequent or Chronic Stomach or Intestinal Problems in the Last 12 Months

Right after he eats, he mentions either a stomachache… But I know stomachaches or stomach issues, and kids can also be related to stress or anxiety. So, it’s possible that there’s some connection there…It’s almost hard to distinguish between the emotional and the physical in terms of when something this major is going on. So, it feels like, almost I’m trying to describe, like, where the physical impacts, the emotional and the emotional impacts, the physical.

—Brielle, mom of 6-year-old boy

Overall, maternal participants reported that 53% (*n* = 264) of the children were experiencing frequent or chronic difficulty with digesting food, including stomach or intestinal problems, constipation or diarrhoea in the last 12 months (Table 4), with differences between the low and high groupings based on the subscales (Table 5; Figure 1). Children in the high Healthcare PSA group (*n* = 252) had twice the odds (aOR: 2.01, *p*-value < 0.001, 95% CI: 1.39–2.92) of reporting stomach problems in the last 12 months as compared to children in the low group (*n* = 245) (Figure 1) when controlling for demographic factors (age, insurance) and household characteristics including ability to afford food, concerns over home eviction, and receiving government assistance (cash assistance/welfare, SNAP, WIC, EBT cards, free or reduced breakfast or lunch at school), and family resilience (Table 5). Children exposed to more frequent Legal PSA had 1.8 times the adjusted odds (aOR: 1.77, *p*-value < 0.003, 95% CI: 1.21–2.59) of experiencing stomach or intestinal problems when controlling for covariates (Figure 1). Stomach problems in the Economic PSA subscale model were not significant (Figure 1).

#### 4.3.2 | Chronic Pain Including Headaches

She has been to the school nurse and…she has had a lot of complaints of abdominal pain, which was something we were supposed to be getting addressed through the allergy and asthma doctor, but my, that’s been a conflict. My ex-husband doesn’t want her to go, and I don’t know why, he actually cancelled her appointment. So, I’m not able to, you know, dive more into what’s going on.

—Candace, mom of 6-year-old girl

Over half (57.8%) of the children were reported to have repeated or chronic physical pain including headaches or other back or body pain in the prior 12 months by maternal participants (Table 4). Children exposed to more frequent Healthcare PSA had 2.2 times the adjusted odds (*p*-value < 0.001, 95% CI: 1.50–3.24) of experiencing chronic pain as compared to children in the low Healthcare PSA group when controlling for demographic and social characteristics (Figure 1). In the Economic PSA model, children exposed to more frequent Economic PSA had 1.6 times the adjusted odds (aOR: 1.60, *p*-value < 0.014, 95% CI: 1.10–2.32) of experiencing chronic pain in the past 12 months (Figure 1). Chronic pain was not significant in the Legal PSA model.

### 4.4 | Mental Health Conditions

#### 4.4.1 | Ever Diagnosed With Depression

My son is experiencing, you know, depression and anxiety, but…he doesn’t feel safe around his father, right?

—Lola, mom of three children, including 14-year-old boy

Over one-third (39.2%; *n* = 195) of children in our sample had ever been diagnosed with depression (Table 4). For children who had ever received a diagnosis of depression, 79% (*n* = 154) currently have depression, with mothers reporting that their child’s depression was mild (26.7%; *n* = 41), moderate (50.7%; *n* = 78) or severe (22.7%; *n* = 35). In the Healthcare PSA model, children exposed to more frequent Healthcare PSA had 1.8 times the adjusted odds (1.80, *p*-value < 0.008, 95% CI: 1.17–2.77) of ever being diagnosed with depression when controlling for covariates (Table 5; Figure 1) as compared to children with low exposure. Children in the high Economic PSA group (aOR: 1.57, *p*-value < 0.03, 95% CI: 1.03–2.39) and high Legal PSA group (aOR: 1.60, *p*-value < 0.037, 95% CI: 1.03–2.47) had approximately 1.6 times the adjusted odds of having ever been diagnosed with depression as compared to children in the low group (Figure 1).

#### 4.4.2 | Ever Diagnosed With Anxiety

They struggle…the girls have…been diagnosed with PTSD. With anxiety. They struggle, the [supervised] visits are hard.

—Meg, mom of 4 children, including 3 girls ages 10, 9, and 7

Nearly two-thirds (73%; *n* = 363) of children had ever been diagnosed with anxiety (Table 4). Of those children ever diagnosed, mothers reported that 91.2% currently have anxiety, and mothers reported that their child’s anxiety was mild 20.0% (*n* = 66), moderate 50.9% (*n* = 168) and severe for 29.1% (*n* = 96) for these children. Ever being diagnosed with anxiety was significant in the adjusted Healthcare PSA model and the adjusted Legal PSA subscale models (Table 5; Figure 1). Children in the high Healthcare PSA group were associated with over two times adjusted odds (aOR: 2.13; *p*-value < 0.001, 95% CI: 1.38–3.30) of having ever been diagnosed with anxiety (Table 5; Figure 1). Children in the high Legal PSA group were associated with an adjusted odds of 2.28 (*p*-value < 0.001, 95% CI: 1.46–3.55) of having ever been diagnosed with anxiety as compared to children in the low Legal PSA group (Figure 1; Table 5). Ever being diagnosed with anxiety was significant in the unadjusted Economic PSA model (OR: 1.63, *p*-value < 0.016, 95% CI: 1.10–2.44); however, once controlling for covariates there was no significant difference among the low and high groups for the Economic PSA model (Table 5).

#### 4.4.3 | Unmet Mental Health Needs

He actively sabotages and prevents them from being around or seeing therapists…he causes a lot of friction, contention, resistance, not showing up to intake appointments with [son] when it’s his parenting time. He’s specifically wanted to keep [son] out of therapy.

—Claudina, mom of two children, including a 13-year-old boy

It was not possible to obtain mental healthcare for 17.5% (*n* = 87) of the children as reported by maternal participants. Inequities existed for children grouped into the low and high groups based on their score on the Healthcare PSA subscale. For children in the high Healthcare PSA group, the adjusted odds of reporting that it was not possible to obtain mental health care were 2.72 times (*p*-value < 0.001, 95% CI: 1.60–4.63) as compared to the low Healthcare PSA group when controlling for covariates (Table 5; Figure 1). Being in the high-exposure Legal PSA group was associated with 1.67 times the odds of it not being possible to obtain mental healthcare; however, the adjusted OR was not significant accounting for demographic and social characteristics (Table 5; Figure 1). Inability to obtain mental healthcare was not significant in the adjusted models for Economic PSA.

### 4.5 | Flourishing

These children have been very severely impacted by the father’s…alcoholism, and his abusive behaviors… In the addiction recovery for families there is also an approach. They don’t call it flourishing. They call it recovery and healing…it’s a framework of surrounding children with supportive, loving, caring adults and, by the way, it only needs to be one…I seek to be that person, but I also really try to have them be around other people…Help them, understand their own body, put their own body and their own mind first, to set boundaries. To speak up, just say what they need to identify their needs and to speak up to people who they can trust, who will meet their needs, get them involved in activities, social networks, social support.

—Georgia, mom of three children, including a 15-year-old girl

Overall, only one in five children (20.5%) met the criteria for flourishing. In the unadjusted models, children in the high Healthcare PSA group were 44% less likely (OR: 0.56, *p*-value < 0.010, 95% CI: 0.36–0.87) to be considered flourishing as compared to children in the low Healthcare PSA model (Table 5; Figure 1). Once adjusting for covariates, the adjusted Healthcare PSA model for flourishing was no longer significant when including family resilience, which was protective. The Economic and Legal PSA subgroup models were not significant for flourishing in the unadjusted or adjusted models.

## 5 | Discussion

This study provides significant evidence of the association between maternal exposure to post-separation abuse and coercion perpetrated by co-parents and adverse child health outcomes for children ages 6–17. Prevention of maternal exposure to IPV and coercive control, including following separation from an abusive partner, is essential to promote child health. Of particular importance, we found strong associations between more frequent exposure to coercive behaviours perpetrated by co-parents and as measured by the Healthcare PSA subscale and all of the child health outcomes we examined. Based on exposure frequency, we found that children exposed to more frequent Healthcare PSA behaviours were 2.7 times more likely for their mothers to report that it was not possible to obtain mental healthcare for their child as compared to children in the low Healthcare PSA group.

These children faced dual burdens: both greater adjusted odds of having received a mental health diagnosis of depression and anxiety and experiencing somatic symptoms of chronic pain and stomach problems in the last 12 months, and their mothers reporting it was not possible to obtain mental health care for them after controlling for demographic and social covariates. These findings are consistent with data from the Fragile Families Dataset where children exposed to maternal IPV victimization had greater rates of unmet health needs (King and Khanijahani 2020), after controlling for sociodemographic characteristics, health insurance status, low birthweight and child abuse and neglect. Among that sample (*N* = 2248), 56% of IPV-exposed mothers were separated from their child’s father. Our study can extend these findings and provide potential insight into mechanisms—and perpetrator behaviours—identified in the items on the HELP-T that may link children’s health care needs and IPV exposure.

These findings advance emerging knowledge at the intersection of IPV, coercive control and paediatric health in several important ways. First, this study is among the first to quantitatively link specific abusive or coercive perpetrator tactics, as measured by the HELP-T subscales, to child health outcomes. Physical IPV exposure alone is insufficient to explain the association of IPV and its harms to children. This study advances the field towards identifying the coercive mechanisms that may contribute to adverse child health outcomes, and will be particularly useful to practitioners in family court and child welfare contexts. Second, by focusing explicitly on the post-separation period, this study addresses a critical gap in the IPV and child health literature. Most existing research captures IPV during cohabitation or an intact parental relationship or does not differentiate between the types of abuse or coercive control that occur following parental separation. These findings suggest that post-separation abuse and coercive control should be viewed as a driver of adverse child health outcomes, independent of the socioeconomic hardships for mothers and children that often accompany separation/divorce. More frequent exposure to PSA is associated with greater harm to children’s health. Third, this study provides preliminary evidence that the HELP-T may be a useful clinical and forensic tool for identifying children at elevated risk of unmet health needs in the context of post-separation abuse, with implications for health care professionals, child welfare systems, and family courts. Together, these contributions provide a foundation for future research to develop targeted interventions and inform policy and legal approaches that centre children’s health needs within family court contexts.

Examining functional difficulties in the past 12 months provides strong supportive evidence of the associations between exposure to higher frequencies of post-separation abuse behaviours and its association with children’s somatic symptoms such as stomach aches and headaches, in that these outcomes were time-bound to the same time frame that the behaviours in the HELP-T. We found that children exposed to more frequent Healthcare PSA behaviours had twice the adjusted odds of experiencing stomach pain in the last 12 months compared to children in the low-exposure group, after controlling for child age, insurance, household ability to afford food, concerns over eviction, receiving government assistance or welfare, and family resilience. Children of mothers exposed to more frequent Legal PSA behaviours had 1.7 times the odds of experiencing stomach or intestinal problems. Functional difficulties of stomach or intestinal problems and chronic pain, including headaches, are consistent with the literature on traumatic stress in children (Fox and Shonkoff 2012).

We did not find a significant association among the low and high groups of the HELP-T subscale for flourishing. Only 19.5% of our overall sample met the criteria for flourishing. Given the low rates of flourishing overall in our sample, it is possible that we could not detect differences among exposure groups or that flourishing is impacted by constructs other than what is measured by the HELP-T.

Across all adjusted models, Family Resilience was protective. This finding is important for prevention efforts, as fostering close connections between children and their non-offending caregivers can help ameliorate the negative health consequences of IPV exposure. It is consistent with the science showing the importance of safe, supportive and nurturing relationships (Bethell et al. 2022; Gillespie 2024; Robinson et al. 2016). Protective, non-offending parents also need systemic support and reduced barriers to promoting care for their children. This is an area for future research and much needed intervention in practice. Specifically, exploring how family court professionals could incorporate child health promotive factors and mitigate PSA tactics within parenting time orders is an important area for future research and practice.

We did not administer a scale for maternal depression and anxiety, although women often experience mental health consequences from abuse. Maternal anxiety and depression may also be linked with children’s depression and anxiety in the context of childhood exposure to IPV (Silva et al. 2021). This is an important exploration in future studies in order to examine the multifaceted pathways through which perpetrator tactics post-separation impact both maternal and child health. To the extent that maternal mental health mediates child health outcomes, it is essential that associations between maternal and child health in this context be understood as consequences of perpetrator behaviour and tactics, with appropriate interventions targeting perpetrator behaviour driving distress. Abusive co-parents who harm mothers are also harming their children. We hypothesise that family court interventions that reduce or eliminate exposure to PSA are likely to improve both maternal and child health simultaneously.

These findings from this study have important implications for practice and policy and for efforts that seek to reduce and prevent violence exposure. These findings can inform interventions to improve health care and health care access for children exposed to IPV. In particular, given the overlap conceptually of medical neglect with Healthcare PSA, the HELP-T can help family court and child welfare professionals and health care professionals identify perpetrator tactics that may prevent children from accessing needed care. Reducing exposure to post-separation abuse for children would, most likely, reduce unmet mental health needs and improve health outcomes, thus reducing health disparities for children exposed to IPV and post-separation abuse. Addressing the impact of IPV and post-separation abuse on children’s health through policy is an important public health intervention. Research on policy approaches to address and mitigate childhood exposure to IPV is scarce. As of February 2022, only 31 states had laws related to children witnessing IPV (Reott et al. 2025), and only one of these mandated a period of supervised parenting for perpetrators of IPV. Laws aimed at protecting children from exposure to IPV by statute serve several important functions including providing general deterrence, signalling societal unacceptability of these behaviours and offering relief to survivors (Reott et al. 2025). Several states have moved to or are considering statutory presumptions of 50/50 joint custody; however, this study provides evidence that more nuanced approaches and fact finding to examine children’s health care needs in the context of post-separation abuse are warranted so that parenting plans can be tailored to individual children’s and families’ needs. Examining the association of these policies with child and family health and well-being is an important future direction for research.

### 5.1 | Limitations

This study has many strengths, including that it is the first study to our knowledge to quantitatively examine children’s health outcomes in the context of post-separation abuse. However, it also has limitations. First, the study relied on a non-representative sample that is subject to selection bias, and participants whose children were experiencing more significant medical needs may have been more motivated to participate. Second, our sample of participants was mostly white, relatively highly educated, and while geographically diverse, was not racially diverse, nor reflective of the general population of US children. Our sample may not represent the experiences of the most marginalized survivors. The results of our study cannot be generalized to the population of children in the US. This limitation in sampling is likely due to our recruitment strategy that relied on social media and snowball recruitment. To our knowledge from participant feedback, our recruitment flyer was posted and shared online in local social media support pages and groups for single mothers by other participants, and it seems many of these communities were predominantly white, or resulted in predominantly white mothers taking our survey. We did not ask about co-parent race/ethnicity, but this would be helpful data to have in order to conduct more comprehensive analyses using an intersectional lens. Future research should focus on outreach to communities that are specifically geared towards Black women, Native women, immigrant women, and other survivors with minoritized identities. These communities may need more culturally-tailored recruitment and outreach strategies, particularly since we were recruiting for legal systems (family court) involvement, and these women may face additional barriers and vulnerabilities to participating in research and may need additional assurances on safety given histories of structural racism and harm within our court systems.

Third, this is a cross-sectional, observational study and we cannot establish causation between post-separation abuse and children’s health outcomes, nor can we establish temporality between exposure and symptoms or diagnosis. That is, we cannot evaluate the association between when experiences of abuse occurred and when children’s health conditions developed or were diagnosed. It is also possible that children with pre-existing health needs elicit or are more susceptible to these forms of abusive and controlling behaviour following parental separation or divorce. In any case, this study provides significant evidence for a strong association between children’s health needs and exposure to post-separation abuse, and children in these circumstances warrant careful adjudication from courts and intervention from health and legal systems to best meet their needs and mitigate harmful consequences of IPV and PSA exposure. Future research should also seek to establish causation, temporal connections and cross-validate the HELP-T in other diverse, representative samples.

## 6 | Conclusion

In this study, we examine the association of post-separation abuse with children’s unmet mental health needs, special health care needs, functional difficulties and ever diagnoses of depression and anxiety, and flourishing. Children whose mothers experienced greater frequency of post-separation abuse had increased odds of having a special health care need, reporting that it was not possible to obtain mental health care, having functional difficulties such as stomach or intestinal problems and chronic pain, and reporting ever having diagnoses of depression and anxiety. Rates of flourishing were low overall for the sample of children affected by post-separation abuse, but were not significantly different among the low- and high-exposure groups.

This study contributes to the literature on children’s health and health needs in the context of maternal IPV victimization following parental separation, providing unique insights into the phenomenon of post-separation abuse and children’s health. The findings of this study highlight the burden of ill health borne by children whose mothers are exposed to post-separation abuse. Special attention and focus from medical and legal systems are needed to help these children and prevent negative health sequelae from post-separation abuse and coercive control.

## Figures and Tables

**FIGURE 1 |:**
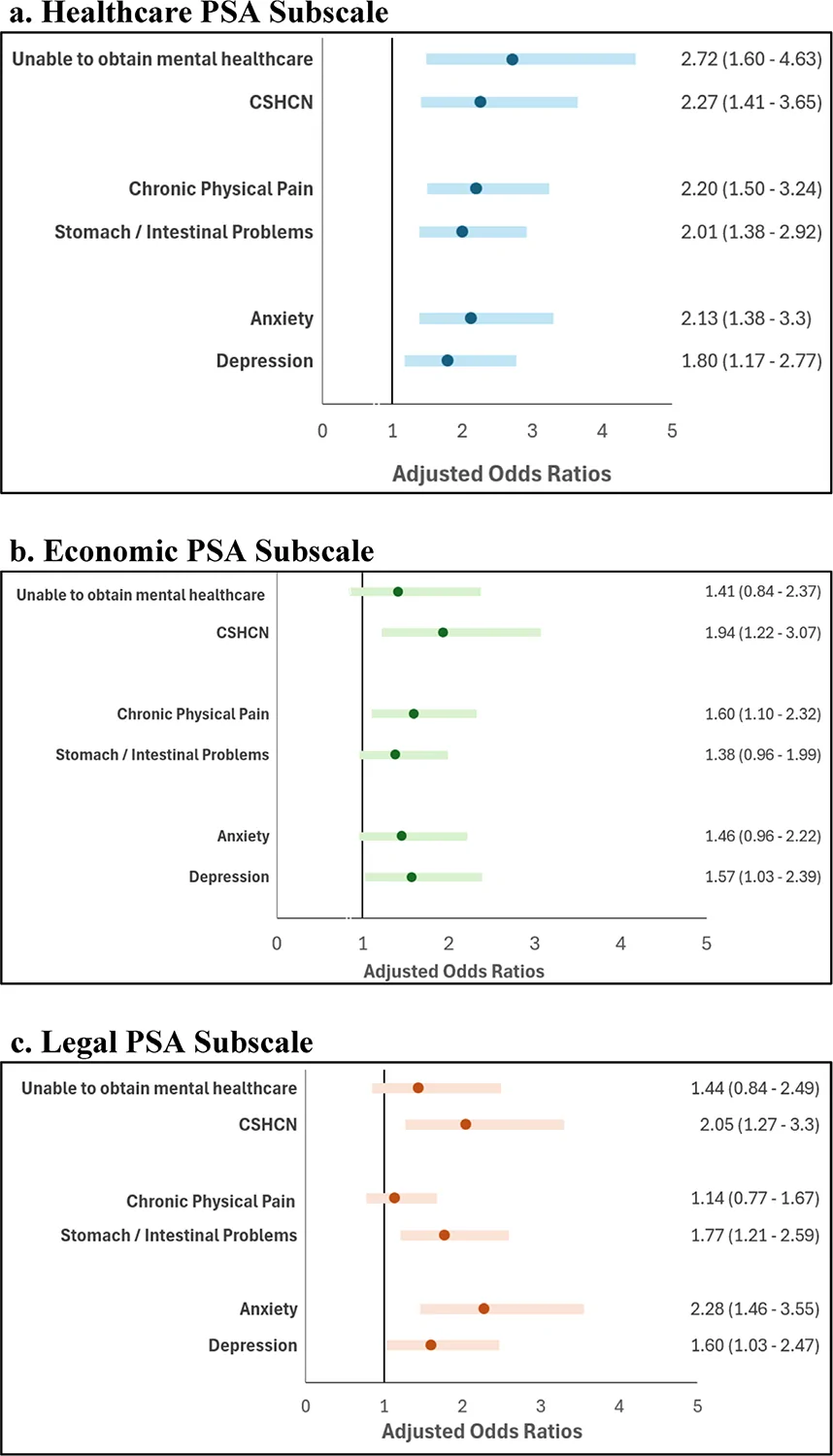
(a–c) Forest plots of adjusted odds ratios for associations between post-separation abuse and coercion exposure by HELP-T subscale and select child health outcomes (*N* = 497). Models adjusted for child age, insurance type (Medicaid, private insurance), ability to afford food, concerns for home eviction, receiving government assistance (cash assistance/welfare, SNAP, WIC, EBT cards, free or reduced breakfast or lunch at school) and family resilience. The low PSA group is the referent (1) for each of the three subscales: Healthcare PSA, Economic PSA and Legal PSA.

**TABLE 1 |:** High and low exposure groupings of HELP-T Scale among participants (*N* = 497).

	Median (IQR)	Low-exposure group (*n*)	High-exposure group (*n*)
Healthcare PSA Subscale	2.30 (1.6–3.0)	245	252
Economic PSA Subscale	3.67 (2.0–4.0)	245	252
Legal PSA Subscale	2.83 (2.0–3.5)	222	275

*Note:* Low-and high-exposure groups defined by a median split for each subscale score.

Abbreviations: IQR, interquartile range; PSA, post-separation abuse.

**TABLE 2 |:** Child and household demographic characteristics (*N* = 497).

Characteristics	*N*	%
Child age (mean, SD)	11.16	3.45
Child sex		
Male	240	48.29
Female	257	51.71
Child ethnicity: Hispanic origin	59	11.87
Child: race		
White alone	422	84.91
Black or African American alone	14	2.82
Two or more races	50	10.06
Other	11	2.21
Health insurance		
Medicaid	144	29.0
Private	318	64.0
Other government insurance	20	4.0
Maternal employment		
Employed full time	296	59.56
Employed part time	104	20.93
Not employed but looking for work	38	7.65
Not employed and not looking for work	32	6.44
Other	27	5.43
Household ability to afford food (past 12 months)		
Always afford to eat good nutritious meals	209	42.05
Always afford enough to eat but not always the kinds of food we should eat	182	36.62
Sometimes we could not afford enough to eat	81	16.30
Often we could not afford enough to eat	25	5.03
Worried about being evicted		
Always	45	9.05
Usually	46	9.26
Sometimes	108	21.73
Rarely	87	17.51
Never	211	42.45
Government assistance during past 12 months		
Cash assistance	84	16.90
SNAP	147	29.58
Free or reduced lunch at school	220	44.27
WIC	25	5.03
EBT	114	22.94
Family resilience	265	53.32

**TABLE 3 |:** Descriptive data on maternal report of IPV exposure from co-parent (*N* = 497).

	Ever		Last 12 months	
Composite Abuse Scale—Short Form Item (CAS-SF)	*n*	%	*n*	%
Physical IPV				
Shook, pushed, grabbed or threw me	347	70%	48	10%
Used or threatened to use a knife or gun or other weapon to harm me	167	34%	27	5%
Threatened to harm or kill me or someone close to me	230	46%	64	13%
Choked me	134	27%	18	4%
Hit me with a fist or object, kicked or bit me	186	37%	24	5%
Confined or locked me in a room or other space	178	36%	26	5%
Sexual IPV				
Made me perform sex acts that I did not want to perform	293	59%	37	7%
Forced or tried to force me to have sex	272	55%	42	8%
Psychological abuse				
Tried to convince my family, children or friends that I am crazy or tried to turn them against me	479	96%	378	76%
Followed me or hung around outside my home or work	316	64%	162	33%
Harassed me by phone, text, email or using social media	438	88%	361	73%
Told me I was crazy, stupid or not good enough	481	97%	336	68%
Kept me from seeing or talking to my family or friends	339	68%	106	21%
Kept me from having access to a job, money or financial resources	350	70%	194	39%
Blamed me for causing their violent behaviour	411	83%	228	46%
Made comments about my sexual past or my sexual performance that made me feel ashamed, inadequate of humiliated	357	72%	128	26%

*Note:* CAS-SF items from Ford-Gilboe et al. (2016) used with permission.

Abbreviation: IPV, intimate partner violence.

**TABLE 4 |:** Select child health outcomes among HELP-T subscale groups.

	Healthcare PSA, *n* (%)		Economic PSA, *n* (%)		Legal PSA, *n* (%)	
	Low (*n* = 245)	High (*n* = 252)	Low (*n* = 245)	High (*n* = 252)	Low (*n* = 222)	High (*n* = 275)
CSHCN	176 (71.8)	216 (85.1)***	178 (72.7)	214 (85.9)**	162 (73.0)	230 (83.6)**
Functional difficulties						
Stomach/intestinal problems	109 (44.5)	155 (61.5)***	120 (49.0)	144 (57.1)	101 (45.5)	163 (59.3)**
Chronic pain	118 (48.6)	169 (67.1)***	125 (51.0)	162 (64.3)**	122 (55.0)	165 (60.0)
Mental health conditions						
Depression	80 (32.7)	115 (45.6)**	78 (31.8)	117 (46.4)**	75 (33.8)	120 (43.6)*
Anxiety	161 (65.7)	202 (80.2)***	167 (68.2)	196 (77.9)*	144 (64.9)	219 (79.6)***
Unmet mental healthcare needs						
Not possible to obtain mental healthcare	25 (10.2)	62 (24.6)***	35 (14.3)	52 (20.6)	30 (13.5)	57 (20.7)*
Flourishing	62 (25.1)	40 (15.9)**	55 (22.5)	47 (18.7)	51 (23.0)	51 (18.6)

*Note:* Chi-square tests (*χ*^2^) were used to compare low- and high-exposure groups for each HELP-T subscale.

Abbreviations: CSHCN, children with special health care needs; PSA, post-separation abuse and coercive control.

* *p* < 0.05,

** *p* ≤ 0.01,

*** *p* ≤ 0.001.

**TABLE 5 |:** Adjusted and unadjusted association of select child health outcomes among HELP-T subscale high and low groups.

	Healthcare PSA		Economic PSA		Legal PSA	
	OR	aOR	OR	aOR	OR	aOR
CSHCN	2.36 (1.50–3.69)***	2.27 (1.41–3.65)***	2.12 (1.35–3.31)***	1.94 (1.22–3.07)**	1.89 (1.22–2.92)**	2.05 (1.30–3.30)**
Functional difficulties						
Stomach/intestinal problems	1.99 (1.39–2.85)***	2.01 (1.39–2.92)***	1.39 (0.98–1.98)	1.38 (0.96–1.99)	1.74 (1.22–2.49)**	1.77 (1.21–2.59)**
Chronic pain	2.19 (1.52–3.15)***	2.20 (1.50–3.24)***	1.73 (1.21–2.48)**	1.60 (1.10–2.32)*	1.23 (0.96–1.76)	1.14 (0.77–1.67)
Mental health conditions						
Depression	1.73 (1.20–2.49)**	1.80 (1.17–2.77)**	1.86 (1.29–2.67)***	1.57 (1.03–2.39)*	1.52 (1.05–2.19)*	1.60 (1.03–2.47)*
Anxiety	2.11 (1.40–3.17)***	2.13 (1.38–3.30)***	1.63 (1.20–2.44)*	1.46 (0.96–2.22)	2.12 (1.42–3.17)***	2.28 (1.46–3.55)***
Unmet mental healthcare needs						
Not possible to obtain mental healthcare	2.87 (1.74–4.75)***	2.72 (1.60–4.63)***	1.56 (0.97–2.50)	1.44 (0.88–2.37)	1.67 (1.03–2.71)*	1.41 (0.84–2.37)
Flourishing	0.56 (0.36–0.87)**	0.65 (0.41–1.05)	0.79 (0.51–1.22)	0.85 (0.53–1.35)	0.76 (0.49–1.18)	0.93 (0.57–1.50)

*Note:* Unadjusted models estimated the bivariate association between each PSA subscale group and child health outcome. Adjusted logistic regression models included a common set of covariates including child age, insurance type (Medicaid, private insurance), ability to afford food, concerns for home eviction, receiving government assistance (cash assistance/welfare, SNAP, WIC, EBT cards, free or reduced breakfast or lunch at school) and family resilience. Adjusted models were run on a total sample size of *N* = 497. Pseudo *R*^2^ results for each logistic regression ranged from 0.04 to 0.19 (Healthcare PSA), 0.03 to 0.19 (Economic PSA) and 0.04 to 0.19 (Legal PSA), indicating acceptable to moderate fit; depression demonstrated the strongest model fit across each subscale model (pseudo *R*^2^ = 0.19). The low PSA group is the referent (1) for each of the three subscales: Healthcare PSA, Economic PSA and Legal PSA.

Abbreviations: aOR, adjusted odds ratio; CSHCN, children with special health care needs; OR, odds ratio; PSA, post-separation abuse and coercive control.

* *p* < 0.05,

** *p* ≤ 0.01,

*** *p* ≤ 0.001.

## Data Availability

Research data are not shared.
